# Chromosome-Level Genome Assemblies of *Darevskia mixta* and *Darevskia valentini*: A Platform for Studying Parthenogenesis in Caucasian Rock Lizards

**DOI:** 10.3390/ani16203311

**Published:** 2026-10-21

**Authors:** Ekaterina M. Dvorianinova, Elizaveta A. Ivankina, Vitaly I. Korchagin, Andrey A. Vergun, Irena A. Martirosyan, Marine S. Arakelyan, Anastasiya E. Girnyk, Alexey A. Dmitriev, Alexey P. Ryskov

**Affiliations:** 1 Laboratory of Genome Organization, Institute of Gene Biology of the Russian Academy of Sciences, 119334 Moscow, Russia; sigova1567@gmail.com (E.A.I.); vitaly_korchagin@rambler.ru (V.I.K.); vermand@mail.ru (A.A.V.); irena-m@yandex.ru (I.A.M.); nasstenochka@mail.ru (A.E.G.); ryskov@mail.ru (A.P.R.); 2 Laboratory of Comparative Genomics and Transcriptomics, Engelhardt Institute of Molecular Biology, Russian Academy of Sciences, 119991 Moscow, Russia; alex_245@mail.ru; 3 Department of Biochemistry, Molecular Biology and Genetics, Moscow Pedagogical State University, 119435 Moscow, Russia; 4 Research Institute of Biology, Yerevan State University, Yerevan 0025, Armenia; arakelyanmarine@ysu.am

**Keywords:** *Darevskia* lizards, parthenogenesis, *Darevskia mixta*, *Darevskia valentini*, genome assembly, population genetics

## Abstract

*Darevskia* lizards attract the attention of many biologists due to the emergence of parthenogenetic species within the genus. In this study, we present chromosome-level genome assemblies of *Darevskia mixta* (Méhely, 1909) and *Darevskia valentini* (Boettger, 1892), two species whose distant ancestors hybridized and formed a parthenogenetic taxon. Genomic DNA was sequenced using the PacBio platform. Primary genome assemblies were generated from ~60 Gb (*D. mixta*) and ~35 Gb (*D. valentini*) of the processed PacBio data using HiFiasm and reference-scaffolded. The final genome sequences of *D. mixta* and *D. valentini* each span ~1.4 Gb, comprising 19 (18 autosomes and the Z chromosome) and 20 (18 autosomes and the Z and W chromosomes) scaffolds, with N50 scaffold values of 91.1 and 97.5 Mb, respectively. Both genome sequences contain ~42% repeats and encode ~41 thousand predicted genes. The assemblies share major structural similarity, but feature a number of inversions and translocations relative to each other. The obtained assemblies represent the first quality platform for unraveling the genetic features associated with the emergence and maintenance of parthenogenesis in *Darevskia* lizards.

## Simple Summary

Some animals can procreate without a mate, producing offspring that are genetic copies of their mother. This unusual form of reproduction is called parthenogenesis. It is rare among vertebrates, but occurs in lizards of the genus *Darevskia* native to the Caucasus Mountains. In this group, several parthenogenetic species arose when pairs of different sexual species interbred in the distant past. One example is *Darevskia armeniaca*, whose parents are *Darevskia mixta* and *Darevskia valentini*. Although parthenogenesis in *Darevskia* draws constant attention, the genetic features that make it possible remain poorly understood. Moreover, reliable resources for genomic research on parthenogenesis in *Darevskia* remain unavailable. In this study, we read long stretches of DNA from *D. mixta* and *D. valentini* and, using a reference genome of a related species, assembled each genome into a full set of chromosomes. The comparison revealed substantial structural similarity between the two genome assemblies, along with several rearrangements. This first genomic resource can help in the study of how parthenogenesis arose in *Darevskia* lizards and identify the genes and chromosome regions involved in this reproductive switch. It also provides a foundation for tracing sex chromosome evolution and establishing relationships among these lizard groups.

## 1. Introduction

*Darevskia* lizards are small reptiles that have garnered scientific interest due to their unusual reproductive biology [1,2]. The genus is remarkable for comprising both bisexual and parthenogenetic species, which currently coexist in nature [3]. The unisexual lineages arose from several independent interspecific hybridizations between bisexual parental lineages within the genus, so it is possible to describe the parthenospecies as F1 interspecific hybrids [4]. Remarkably, all parthenogenetic *Darevskia* species owe their origin to only four sexual taxa [5,6]. Despite years of research, it is still unknown which molecular mechanisms underlie this exceptional ability to hybridize with the emergence of parthenogenetic offsprings.

Unlike the molecular pathways, the cellular mechanism of *Darevskia* parthenogenetic taxa formation has been established recently. In oocytes of a mature viable F1 hybrid, premeiotic endoreplication doubles the genetic material [2,7]. Thus, homologous chromosomes can properly pair and undergo recombination. Finally, a diploid egg cell is formed. The knowledge gap in the understanding of this process concerns two main questions. What is the molecular trigger of endomeiotic replication? Why is the process restricted only to certain parental species? These questions can be addressed using omics data. Genome sequences are particularly useful tools for identifying candidate regions that may be associated with the transition to parthenogenesis.

The first genome assembly of a lizard was obtained for the green anole [8]. Then, many other reptilian genomes followed [9,10,11,12,13,14,15,16,17,18,19]. These early assemblies were still rather fragmented. However, the advancement of sequencing technologies led to improvement in the quality and quantity of the obtained data. Recently, telomere-to-telomere (T2T) or near-T2T reptile genome sequences were reported [20,21,22]. The high accuracy and completeness of the obtained assemblies provide a rich source for extensive and reliable studies relying on genomic data [23,24,25].

High-quality genome assemblies of parthenogenetic species, coupled with transcriptomic data or used in comparative genomic analysis, are brilliant sources of knowledge on the unisexual mode of reproduction, particularly in studies that include parent–hybrid (father/mother/progeny) comparisons [26,27,28]. Using full genome sequences, one can trace the evolution of different genomic regions. For instance, Lv et al. studied the synteny and specific repeat distribution of a flowerpot snake genome to divide chromosomes into three subgenomes and suggest their origin [29]. Another example is a study by Warren et al. [30]. The authors demonstrated a hybrid origin of the Amazon molly (*Poecilia formosa*) genome, comparing it with genome assemblies of its parental species. The hybrid nature of the species’ genome was also proven with the calculated heterozygosity rate. In addition, linkage analysis confirmed the “frozen” state of the *P. formosa* genome and the absence of recombination. Thus, high-quality genome sequences serve as a platform for subsequent extensive studies of parthenogenesis and its related aspects. In light of this fact, creating accurate genome assemblies is essential to enable further relevant research.

For *Darevskia* lizards, a comprehensive genomic resource is still absent. This gap significantly complicates further research on the aspects of parthenogenesis. In our previous study, we assembled the genome of *Darevskia valentini* (Boettger, 1892) [31], one of the parental species contributing to parthenogenetic taxa formation. Despite the extensive groundwork, the assembly remained fragmented, as it was based solely on 10x linked-reads. However, genome assemblies of both parthenogenetic lineages and the related parental species could provide valuable information on the reasons behind parthenogenesis emergence.

This study aimed to generate and compare high-quality genome assemblies of *D. valentini* and *Darevskia mixta* (Méhely, 1909). This pair of sexual taxa underwent possibly multiple hybridization events and produced *Darevskia armeniaca*, a parthenogenetic *Darevskia* species [4,6,32]. Thus, the genomes of *D. mixta* and *D. valentini* are a suitable model to identify molecular features that could have enabled parthenogenesis in their hybrid offspring. We present reference-scaffolded chromosome-level genome assemblies for both parental species and conduct a primary comparative analysis between them. Our work creates the first framework for further targeted investigation into genomic regions potentially associated with parthenogenesis in *Darevskia*.

## 2. Materials and Methods

### 2.1. Sample Collection and Surgical Procedures

To obtain DNA, blood samples (stored in EDTA buffer at −20 °C) of three male *D. mixta* individuals and one female *D. valentini* individual were used. *D. valentini* sample was collected in Armenia outside of protected areas [31]. *D. mixta* samples were collected from the Borjomi population in Georgia outside of the protected areas during expeditions in 1998–2000 and kindly provided to the Laboratory of Genome Organization (IGB RAS) by Ilya Darevsky (Zoological Institute of Russian Academy of Sciences). *D. mixta* and *D. valentini* are not listed under CITES (https://cites.org/, accessed on 1 May 2026).

### 2.2. DNA Extraction and Sequencing

Total genomic DNA was extracted from blood samples using the standard phenol–chloroform method [33] and dissolved in TE buffer (pH = 8.0). DNA purity was assessed via the measurement of light absorbance (A230/A260/A280 ratios) on a Genesys UV-Vis spectrophotometer (Thermo Fisher Scientific, Waltham, MA, USA). Only DNA samples with A260/A280 ratio ≥ 1.8 and A260/A230 ratio ≥ 2.0 were taken for sequencing and were analyzed for integrity with 0.5% agarose gel electrophoresis. For one *D. mixta* and one *D. valentini* sample, long-read library preparation and PacBio high-fidelity (HiFi) sequencing on PacBio Sequel II/IIE (Pacific Biosciences, Menlo Park, CA, USA) were performed by Novogene (Beijing, China). Three DNA samples of *D. mixta*, including the specimen sequenced on PacBio, were used to produce short-read libraries (150 PE, Illumina TruSeq DNA PCR Free Sample Prep protocol) and sequenced by Novogene (Beijing, China) on Illumina NovaSeq 6000 (Illumina, San Diego, CA, USA).

### 2.3. Data Processing and Genome Assembly

PacBio reads in fastq format were cleaned from the adapter sequences with Porechop 0.2.4 (https://github.com/rrwick/porechop, accessed on 1 May 2026) and filtered by length (a minimum of 10 kb) and quality (no lower than Q15) with Seqkit 2.8.2 seq [34]. Raw Illumina reads were processed with Trimmmomatic-0.38 [35] to remove adapter sequences and low-quality bases (TRAILING:28, SLIDINGWINDOW:4:20, MINLEN:50).

Primary genome assemblies were generated with HiFiasm 0.25.0-r726 [36] (for *D. mixta* and *D. valentini*) and Verkko v2.2.1 (only for *D. mixta*) [37] from doubled volumes of PacBio reads. Contamination control was performed with Kraken 2.1.6 [38]. To extract taxids (taxonomic IDs) matching other species than bacteria, grep (“-v”, “-P” options) and awk commands were used. Contigs corresponding to *Darevskia* were extracted from the Kraken output according to the filtered taxon IDs. For *D. mixta*, the cleaned assembly was polished with trimmed Illumina reads using Pilon 1.24 [39] to correct possible sequence errors. QUAST v5.0.2 [40], Seqkit 2.8.2, and BUSCO 5.8.3 (sauropsida_odb10) [41] were used for assembly quality assessment.

To identify the positions of the assembled contigs in a reference assembly, the *Lacerta agilis* L. genome sequence (NCBI Genome, GCA_009819535.1) was used. This species is one of the closest lacertid taxa to *Darevskia*. Whole-genome alignment was performed with LAST 1595 (https://gitlab.com/mcfrith/last, accessed on 1 May 2026) (with options: “-v”, “--sort2 = 3”, “--strands2 = 1”, “--maxseqs = 100” or “--maxseqs = 1000”). Scaffolding was performed with RagTag v2.1.0 [42] using the *L. agilis* genome assembly and manually curated according to the output alignment information of both LAST and RagTag. The final contig start coordinates upon assembly scaffolding, as well as their sizes, positions, and orientations, were taken from the “.agp” file generated by RagTag. In the final scaffold headers, the prefix “NC_” was replaced with “scaffold_”. Telomere sequences were searched with Tidk 0.2.41 (repeat window of 1000) (https://github.com/tolkit/telomeric-identifier, accessed on 1 May 2026).

The quality of the final assembly of *D. mixta* was additionally judged by the consensus quality (QV) score and the percentage of the aligned Illumina data. QV estimation was conducted with Merqury v1.3 [43]. A k-mer database (k = 31) was built using Meryl and the trimmed Illumina reads. The output was passed to merqury.sh along with the final assembly. Samtools 1.10 [44] flagstat was used to calculate the total number of mapped Illumina reads. The total number of bases covered at least once was calculated from bedtools v2.30.0–8-g2fd5181a output piped to an awk command (bedtools genomecov -ibam … -bga 2>/dev/null | awk ‘$4>0 {c+=$3-$2} END {print c+0}’).

Databases of repeat sequences were built with RepeatModeler 2.0.7 (‘-LTRStruct’ option was enabled) [45] and repeats were masked with RepeatMasker 4.1.7 (https://www.repeatmasker.org/, accessed on 1 May 2026) [46]. Gene annotations of the masked assemblies were created with Braker 3.0.8 (‘--softmasking’, ‘--gff3’, ‘--species=lacertidae’) [47], and statistics of the obtained annotation files were calculated with AGAT 1.4.2 [48] and the “grep -c” command. To support the predicted gene models of the *D. valentini* annotation, raw read counts were obtained with PPLine [49] using the RNA-Seq data from Ochkalova et al. [31] (NCBI SRA: SRX12402759, SRX12751488, and SRX15406814). Counts per million (CPM) values were calculated as a raw count divided by the total library size, multiplied by one million.

For single-nucleotide polymorphism (SNP) detection, PPLine [49] was run on the final *D. mixta* genome assembly and Illumina reads of three specimens of this species. The number of SNPs was calculated from the output alignments. Due to the low genome coverage with Illumina reads, a low alternate allele fraction threshold was used in freebayes for SNP detection to retain true heterozygous sites, at the cost of potentially including false-positive calls. Alignment files (in bam format) were passed to freebayes v1.3.1-dirty [50] (the following options were used: “--ploidy 2”, “--min-mapping-quality 30”, “--min-base-quality 20”, “--min-alternate-count 2”, “--min-alternate-fraction 0.05”, “--min-repeat-entropy 1”, “--use-best-n-alleles 4”), and the output files were passed to bcftools 1.9 [44] for further filtration (“-i ‘INFO/AF > 0.3 && QUAL>30 && INFO/DP>5 && INFO/DP<30 && INFO/MQM>40 && (strlen(REF)=1 && strlen(ALT)=1)’”, “-m2”, “-M2” options were enabled). To extract SNPs corresponding to the coding regions, bcftools view was run on the obtained sorted and indexed vcf files, using the “-R” option and a bed file generated from the *D. mixta* genome assembly annotation (created in this study). To check the Ts/Tv ratio (transition/transversion), the resulting vcf files were passed to bcftools stats with the output piped to an awk command (“awk ‘/^TSTV/ {print “ts:”, $3, “tv:”, $4, “ratio:”, $5}’”).

For runs of homozygosity (ROH) analysis, variant calling was repeated with freebayes using the same options as for SNP detection, except for a higher alternate allele fraction threshold (“--min-alternate-fraction 0.1”), which reduced the number of false heterozygous calls, and without the repeat entropy filter, which was omitted to retain true heterozygous sites within repetitive regions. The resulting vcf files were filtered with bcftools (“-i ‘QUAL>30 && INFO/DP>5 && INFO/DP<30 && INFO/MQM>40 && (strlen(REF)=1 && strlen(ALT)=1)’”, “-m2”, “-M2”). Filtering by allele frequency was excluded to avoid introducing bias in ROH estimates. The filtered files were processed with the bcftools roh module (“-G30”, “--AF-dflt 0.4”). The genomic inbreeding coefficient was calculated for each individual as the total length of autosomal ROH segments divided by the total autosomal assembly length, i.e., the assembly size (1.4 Gb) excluding the Z chromosome (45.3 Mb). Assembly gaps (runs of “N”) were retained in the length calculations. However, their contribution to the autosomal length was negligible.

The genome-wide heterozygosity rate was estimated using the same filtered vcf files that were obtained for the ROH analysis. The number of heterozygous sites was calculated with bcftools view (“-H”, “-i ‘GT=“het”’”). The Z chromosome calls were excluded from the counts. Heterozygosity was calculated as the number of heterozygous autosomal sites divided by the total autosomal assembly length.

To perform synteny analysis, syri 1.7.1 (https://github.com/schneebergerlab/syri, accessed on 1 May 2026) [51] was used. The final assembly of *D. mixta* was aligned to the final assembly of *D. valentini* using minimap2 (‘-ax asm5’, ‘--eqx’) [52]. The alignment was sorted with samtools 1.10 [53], output to bam files, and indexed. Structural variations were detected with syri, and plotting was performed with plotsr 1.1.0 (https://github.com/schneebergerlab/plotsr, accessed on 1 May 2026) [54].

## 3. Results

Nearly 99.94% of PacBio sequencing reads of *D. mixta* and 99.91% of those of *D. valentini* exceeded the length of 10 kb and had a minimum quality of Q15. Using *D. mixta* sequencing data, we developed the protocol for genome assembly and applied it to *D. valentini*. Draft genomes of *D. mixta* and *D. valentini* were assembled from 61.3 Gb and 34.8 Gb of filtered PacBio HiFi reads, with average read lengths of 17.1 kb and 18.0 kb, corresponding to ~40× and ~25× genome coverage (assuming a genome size of ~1.4 Gb), respectively. Primary assemblies of *D. mixta* were generated using HiFiasm and Verkko. Assembly quality was assessed with BUSCO (sauropsida_odb10 dataset) and QUAST (Table 1).

The *D. mixta* genome assembly generated with HiFiasm consisted of 555 contigs and had a size of 1.48 Gb, an L50 of 18, and an N50 of 28.07 Mb. Verkko demonstrated markedly poorer QUAST statistics. The tool assembled a genome of nearly 2.3-fold greater size. It comprised 50,700 contigs and had an N50 of only 0.15 Mb. In addition, the ratio of duplicated orthologues was strikingly high (73.5%), additionally pointing to the presence of several haplotypes. Therefore, only the assembly by HiFiasm was taken for further improvement and analysis.

On ranging contigs with QUAST (Icarus), we observed that nearly 80% of all sequences had a size smaller than 1 Mb. To investigate the origin of these small fragments, we checked the obtained genome assembly for contamination with bacterial sequences using Kraken. Any contigs matching contaminating taxa were removed. QUAST and BUSCO statistics of the cleaned assembly remained largely unchanged (Table 1) except for the number of contigs, which reduced almost by half. This indicated a minor impact of possible contamination on the genome assembly’s quality. To improve the accuracy of the cleaned assembly, we polished it with preprocessed Illumina reads using Pilon (~16× coverage with the trimmed reads).

Then, we analyzed the contribution of contigs to the size of the resulting assembly in the Icarus viewer. We observed that nearly half of all contigs had a small size (smaller than 300 kb) and occupied a negligible proportion of the assembly. Such sequences could represent poorly assembled regions of low coverage and may interfere with further analysis and genome annotation. Therefore, we filtered out contigs smaller than 300 kb with Seqkit. The resulting assembly had a size of 1.40 Gb, an N50 of 30.30 Mb, and an L50 of 17, and consisted of 104 contigs (Table 1).

The *D. valentini* genome sequence was assembled using HiFiasm and filtered from contigs smaller than 300 kb. Since biological contamination was absent according to the Kraken output, filtration from non-target sequences was omitted. QUAST and BUSCO statistics were calculated (Table 2). The resulting genome assembly of *D. valentini* comprised 306 contigs, totaled 1.44 Gb in size, and had an N50 of 11.11 Mb and an L50 of 39 (Table 2).

The obtained contig-level genome assemblies of *D. mixta* and *D. valentini* were aligned to the chromosome-level genome sequence of a close taxon—*Lacerta agilis* L. (NCBI Genome, GCA_009819535.1) (Supplementary Figure S1). The alignment of both assemblies showed a high structural similarity to the reference. To analyze chromosomal positions of contigs from both *Darevskia* genome assemblies, we used the obtained alignment information.

Sex chromosomes are potential sources of structural rearrangements associated with parthenogenesis [2,6,7,55,56]. Therefore, these regions deserve a detailed analysis. *Darevskia* lizards possess a ZW system of sex determination, with homogametic males and heterogametic females [55]. In our study, we used DNA samples of a male *D. mixta* and a female *D. valentini* and obtained genome assemblies of the merged haplotypes within each genome. In the *D. mixta* assembly, we observed five contigs corresponding to the Z sex chromosome of *L. agilis* (NC_046331.1)—ptg000011l_pilon, ptg000052l_pilon, ptg000077l_pilon, ptg000046l_pilon, and ptg000137l_pilon (Supplementary Table S1 and Figure S1a). The alignment showed a high structural similarity of male chromosomes between the analyzed species. In the assembly of *D. valentini*, the sequence of the Z chromosome consisted of many more contigs (Supplementary Table S2 and Figure S1b) than that of *D. mixta* (Supplementary Table S1 and Figure S1a). This feature can be explained by the lower coverage of the *D. valentini* genome with sequencing data (~25× for *D. valentini* vs. ~40× for *D. mixta*). The small W chromosome of *D. valentini* was represented by two contigs—ptg000163l and ptg000194l (Supplementary Table S2 and Figure S1b). Its sequence was highly repetitive.

In general, only small rearrangements were revealed in the contigs from the assembled *Darevskia* genomes compared to the *L. agilis* assembly. For example, we observed an inversion in the ptg000009l_pilon contig (chromosome 3 in the genome of *L. agilis*—NC_046314.1) in the *D. mixta* assembly (Supplementary Figure S1a) and an inversion in the ptg000094l contig (chromosome 5 in the genome of *L. agilis*—NC_046316.1) in the *D. valentini* assembly (Supplementary Figure S1b).

In the analysis of contig positions, we scaffolded the assemblies of *D. mixta* and *D. valentini* with RagTag using the genome sequence of *L. agilis* as a reference due to karyotype conservation in the family Lacertidae [7,57]. Thus, the chromosome-scale structure reported here is inferred rather than empirically determined. The distribution of the telomeric repeat was analyzed across the *D. mixta* and *D. valentini* scaffolds to investigate assembly completeness and accuracy. In the scaffolded chromosome 1 of *D. mixta* (corresponding to chromosome 1 of *L. agilis*—NC_046312.1), the sequence immediately following the first integrated 6.6 Mb contig (ptg000087l_pilon) began with a telomeric repeat (ptg000006l_pilon). Given that telomeric repeats mark chromosomal ends, we partitioned this leading contig from the *D. mixta* chromosome 1 scaffold. Thus, for *D. mixta*, we obtained an assembly of 19 scaffolded chromosomes (and nine unplaced contigs) with an N50 of 91.1 Mb and an L50 of 7. In the obtained assembly, 14 of 19 scaffolds had a high telomeric repeat occurrence at either one (12 scaffolds) or both ends (two scaffolds). The assembly of *D. valentini* consisted of 20 scaffolds and 18 unplaced contigs with an N50 of 97.5 Mb. Eight of these scaffolds had assembled telomeric repeat sequences at both ends. In addition to the obtained *Darevskia* genome assemblies, we analyzed telomeric repeat frequency across the chromosomes of the reference species—*L. agilis*. We observed that the chromosomes of *Darevskia* species contained more assembled telomeric repeats than those of *L. agilis*. This fact pointed at a better resolution of chromosome end sequences in the *Darevskia* assemblies than in the assembly of *L. agilis*.

The final *D. mixta* and *D. valentini* assemblies demonstrated high structural similarity both to each other and to the *L. agilis* assembly, but also showed a number of inversions and translocations (Supplementary Figure S2). The observed structural rearrangements between the genome assemblies of Lacertidae species were already present at the contig level (Supplementary Figure S1). Therefore, none of them should be attributed to possible assembly scaffolding missteps.

For the additional quality assessment of the final *D. mixta* assembly, Illumina reads were mapped. On alignment with BWA, 99.99% of the paired-end reads successfully mapped to the genome assembly, and 99.20% of the assembly bases were covered. The alignment results indicated a high quality of the obtained genome sequence. In addition to the alignment tests, the same Illumina data (~16× genome coverage) was used to estimate assembly accuracy with Merqury. The tool calculates the QV score from the k-mer occurrence in both read and assembly data. Our analysis demonstrated that the obtained genome sequence of *D. mixta* had a QV of 34.1 (may be underestimated due to low genome coverage), corresponding to an error rate of 0.038%. This error rate reflects quite high sequence accuracy and supports the idea of the overall quality of the assembly. Thus, we developed a systematic step-by-step workflow (Supplementary Figure S3) and generated a high-quality genomic resource for a pair of *Darevskia* species that formed a parthenogenetic taxon.

The final *D. mixta* and *D. valentini* genome assemblies were subjected to repeat masking and structural annotation. Running RepeatModeler and RepeatMasker resulted in 41.86% and 42.11% of masked sequences, respectively. Retroelements accounted for 24.36% and 23.64% of the assembly sizes, and long interspersed nuclear elements (LINEs) constituted 16.91% and 16.50% of the total genome sequences, respectively. DNA transposons occupied 6.12% and 6.46% and unclassified repeats accounted for 9.81% and 10.34% of the *D. mixta* and *D. valentini* assemblies, respectively. Gene sequences were predicted with Braker3. The resulting annotation of *D. mixta* comprised 39,886 genes and 62,124 transcripts. For *D. valentini*, 41,692 genes and 64,333 transcripts were predicted. Such high gene numbers are likely attributable to ab initio gene prediction alone with Braker3, without RNA-Seq evidence and the manual curation step that removes redundant models. To provide expression support for the predicted gene models, we mapped the transcriptomic data from Ochkalova et al. [31], obtained from kidney, liver, brain, and ovary tissues of *D. valentini*, to its genome assembly created in this study. Of the 41,692 genes, 12,746 had CPM values above 0.5, with 85.2% of reads uniquely mapped.

Upon assembly annotation, we conducted a search for polymorphisms using Illumina whole-genome sequencing data of three *D. mixta* individuals (~15× genome coverage on average) to roughly estimate the *D. mixta* population diversity. Given the low coverage, variant calling thresholds were adjusted during the calculation of the SNP number, heterozygosity rate, and genomic inbreeding coefficient to balance the retention of true heterozygous sites against the inclusion of false-positive calls. One of the analyzed *D. mixta* samples was the same one that was used for the genome assembly. Analysis of its Illumina data revealed about 97 thousand SNPs (heterozygous or alternative homozygous variants with a VAF (variant allele frequency) of more than 0.3 and a minimum coverage of 5) across the coding regions and 2.6 million SNPs across the whole genome. The observed numbers of alternative and heterozygous variants are plausible, as the created genome assembly represents a single merged haplotype of the diploid *D. mixta*. The alternative and heterozygous SNPs arose primarily from aligning the Illumina data of both haplotypes to this consensus assembly. Analysis of the coding regions of the other two *D. mixta* individuals revealed about 217 and 117 thousand SNPs. At the whole-genome level, we calculated about 6.0 and 3.2 million SNPs for these samples, respectively. To ensure the correctness of the obtained values, transition/transversion ratios were checked. For the whole-genome data of all analyzed samples, the ratio exceeded 1.95, indicating a high accuracy of the analysis. To complement the SNP-based estimates of population diversity, we calculated genome-wide heterozygosity for each sample in an independent analysis. The heterozygosity rate of the individual used for the genome assembly was 0.0019, while the rates for the other two individuals were 0.0023 and 0.0018. To estimate the level of inbreeding, runs of homozygosity (ROH) were analyzed for each individual in the sampled *D. mixta* population. The genomic inbreeding coefficient (FROH) was 0.016 for the individual used for the genome assembly, and 0.296 and 0.178 for the other two individuals. Total autosomal ROH lengths were 22.0 Mb, 401.1 Mb, and 241.3 Mb, respectively. Since ROH detection is limited at low coverage, the calculated values may be underestimated.

**Figure 1:**
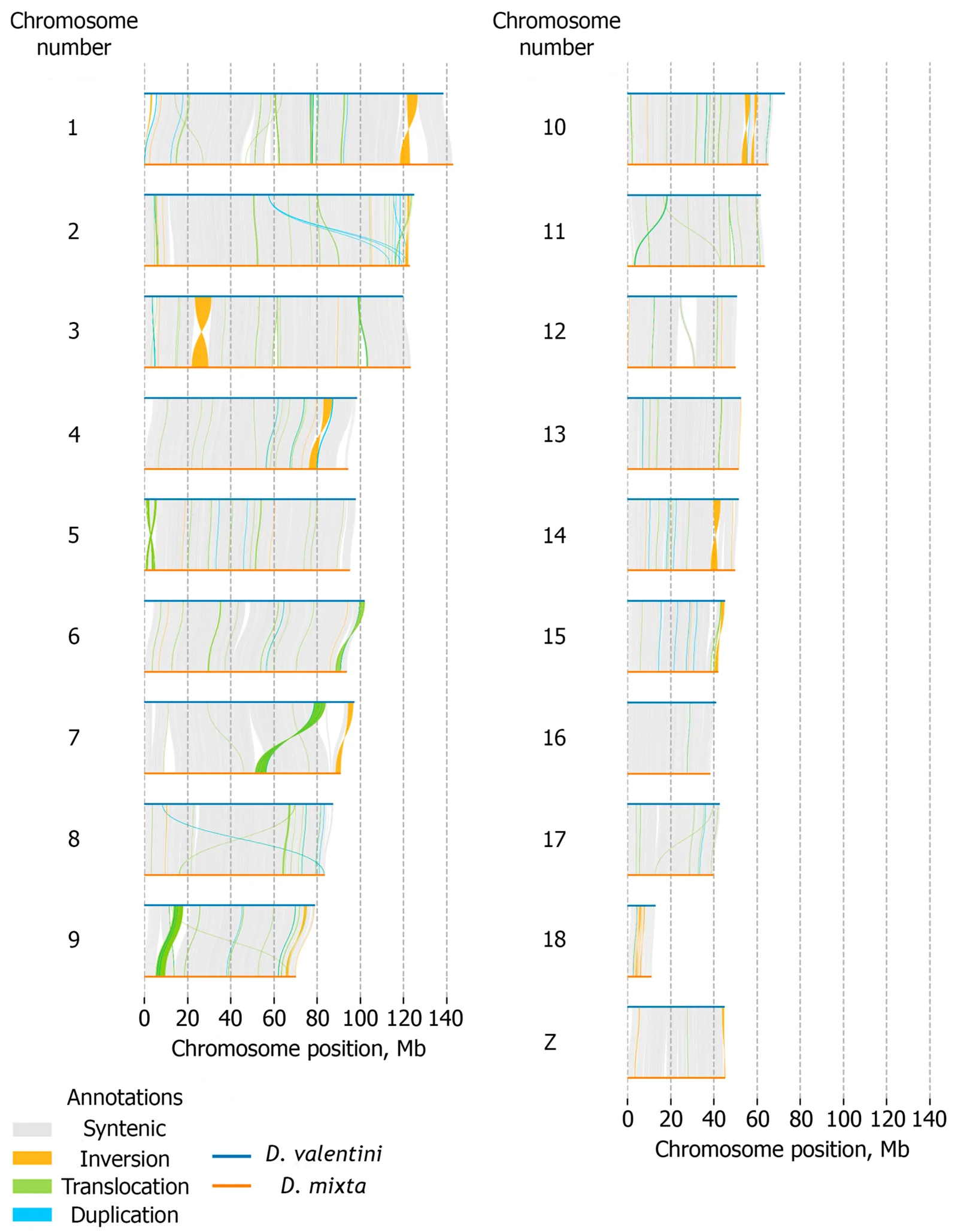
Synteny between homologous chromosomes (vertical axis) of *D. mixta* (blue horizontal lines) and *D. valentini* (orange horizontal lines). Syntenic regions are connected with gray curved lines. Structural rearrangements are represented as curved arcs: orange arcs—inversions; green arcs—translocations; blue arcs—duplications.

Analyzing the degree of similarity between the assembled genomes of *D. mixta* and *D. valentini* is particularly useful in light of possible meiosis disruptions between the genetic material of these species during the emergence of their parthenogenetic offspring. To search for the differences between the *D. mixta* and *D. valentini* assemblies, we performed whole-genome alignment with LAST (Supplementary Figure S4) and conducted synteny analysis (Figure 1).

The assemblies shared high structural similarity, except for several rearrangements. For instance, a number of inversions are evident in the aligned chromosomes, e.g., the ones located in chromosomes 3 and 14 (Supplementary Figure S4). Synteny analysis between homologous chromosomes in the assembled *Darevskia* genomes additionally confirmed large-scale structural conservation. However, multiple inversions and translocations were also detected (Figure 1). Both LAST alignment and synteny analysis revealed the absence of large intrachromosomal genome duplications. The Z chromosomes of the analyzed species were highly syntenic, despite several small inversions.

## 4. Discussion

Parthenogenesis is a rare phenomenon in vertebrate animals [58]. Among amniotes, unisexuality was first identified within the group of Caucasian rock lizards [59]. Within the genus *Darevskia*, parthenogenetic lineages arose several times upon interspecific hybridization between sexual species [32,60]. Until now, the *Darevskia* lizard species have represented one of the most well-studied reptile systems in biogeography, ecology, and population and molecular genetics, but not at the whole-genome level [1]. Of the 40 described *Darevskia* species, seven reproduce parthenogenetically and share the same history of parthenogenesis emergence [1,61]. For two parthenogenetic species, *D. armeniaca* and *D. dahli*, *D. mixta* is an exclusive maternal ancestor; for five parthenospecies, *D. armeniaca*, *D. unisexualis*, *D. uzzelli*, *D. sapphirina*, and *D. bendimahiensis*, *D. valentini* is an exclusive paternal ancestor [6]. In this study, we focused on *D. mixta* and *D. valentini*—the parental species for unisexual *D. armeniaca*. According to Murphy et al. [6], one of the possible reasons limiting the ability of *Darevskia* species to form parthenogenetic hybrids is associated with the sex chromosome system. Therefore, *D. mixta* and *D. valentini* represent the first *Darevskia* model system to study the origins of parthenogenesis at the genomic level. To uncover the molecular aspects of parthenogenesis, genome assemblies of both parthenogenetic species and their corresponding parents are essential. The comparative analysis between the obtained assemblies can reveal genomic features underlying the parthenogenetic mode of reproduction in *Darevskia*. We assembled the genome sequences of *D. mixta* and *D. valentini* and performed an initial synteny analysis between them. Scaffolding to the chromosome level was performed using the *L. agilis* genome assembly as a reference. The choice of *L. agilis* is supported by the highly conserved karyotype within Lacertidae. Srikulnath et al. documented linkage homology between the *L. agilis* genome and other squamates [57], while Spangenberg et al. directly demonstrated chromosomal homology between parthenogenetic *Darevskia* species and their parental lineages using cytogenetic methods [62]. Given this karyotype conservation, contigs of *D. mixta* and *D. valentini* were assigned to chromosomes of *L. agilis*. However, since the chromosome-level assemblies presented in this study are inferred from conserved synteny, contig placement should be empirically validated in future chromatin conformation studies. Therefore, the genomes of *D. mixta* and *D. valentini* constructed here should be considered reference-scaffolded chromosome-level assemblies.

The final genome sequences of *D. mixta* and *D. valentini* have a size of ~1.4 Gb. The assembly of male *D. mixta* comprises 19 scaffolds (18 autosomes and the Z chromosome) and nine unplaced contigs, with an N50 of 91.1 Mb and an L50 of 7. For the final assembly of female *D. valentini*, we achieved generally similar statistics—20 scaffolds (18 autosomes, the Z and W chromosomes) and 18 unplaced contigs, an N50 of 97.5 Mb, and an L50 of 7. Thus, a complete haploid set of chromosomes was obtained for both genomes.

The generated genome assemblies of *D. mixta* and *D. valentini* were searched for repetitive sequences. Reptile genomes are characterized by the high content of repeats, including transposable elements (TEs) [63]. In the assemblies of both *Darevskia* species, ~42% of the sequence was occupied by repeats. The observed values fall within the range of multicopy sequence percentages described in literature data for reptiles [64,65]. Since Squamata exhibit high lineage specificity of TEs [66,67,68], future deeper analysis of repeats in the genomes of *D. mixta* and *D. valentini* and their parthenogenetic progenitor may be useful for tracing the emergence of the formed hybrid genome [25]. However, the current lineages of the chosen *Darevskia* species might have significantly diverged from their ancestral populations that participated in hybridization events. Therefore, the general genome evolution of *D. mixta* and *D. valentini* should also be considered in such an analysis.

In addition to repeat search, gene annotation was performed for the assembled *Darevskia* genomes. Both assemblies contained ~41 thousand genes and ~63 thousand transcripts. These numbers correspond to literature data on reptile gene content predicted with an ab initio method. In *Lacerta viridis* and *Lacerta bilineata*, 38–55 thousand coding sequences and predicted proteins were annotated using ab initio methods and refined to a final number of 22,100–22,500 gene models, each supported with RNA-Seq data during the curation step [69]. A similar pattern was observed for other closely related species *Podarcis raffoneii* and *Podarcis muralis*, where ab initio predictions were combined with transcript and protein evidence to produce final annotations of approximately 24,600 and 20,900 protein-coding genes, respectively [70,71]. *D. mixta* and *D. valentini* annotations are based on raw ab initio predictions without the post-processing steps that retain only transcript-supported genes and merge the redundant models. To partially address this, we mapped the kidney, liver, brain, and ovary transcriptomic data from Ochkalova et al. [31] to the annotated *D. valentini* assembly. About 13 thousand predicted genes had CPM values above 0.5, while ~85% of transcriptomic reads were mapped uniquely. Thus, about a third of the *D. valentini* annotation was supported by transcriptomic reads. However, since transcriptomic data covered only four tissue types, the fraction of supported genes is likely an underestimate. No transcriptomic data currently exist for *D. mixta*, so its annotation could not be assessed in this way. Nevertheless, the obtained annotated chromosome-level assemblies already provide a useful resource. While the coding sequence count requires refinement, the underlying gene models contain protein-coding sequences and can be processed in further research on *Darevskia*.

The chromosomal structure of the assembled *D. mixta* and *D. valentini* genomes was investigated. Most species from the family Lacertidae have 38 acrocentric chromosomes (ploidy of 2*n* = 38) and a ZZ/ZW system of sex determination, with heterogametic females [72,73,74]. The W chromosome in lacertid lizards is highly degenerated [75]. A large ratio of its sequence belongs to repetitive regions. Repeat accumulation is clearly visible in the plot of alignment between the female *D. valentini* and *L. agilis* assemblies (chromosome W—NC_046330.1, *L. agilis*) (Supplementary Figure S2). Meanwhile, the Z sex chromosomes of the analyzed Lacertidae species are structurally conserved and show a similar repeat content to that of the autosomes. The identified striking differences between the structures of the Z and W chromosomes highlight the degenerative state of the latter, confirming the previous observations on lacertid species [75].

Not only sex chromosomes have a significant impact on animal reproduction, but the whole karyotype structure [76]. *Darevskia* hybrid lineages inherited complete haploid chromosome sets from their ancestral species [77]. Presumably, it is the incompatibility of the parental karyotypes that led to the switch to parthenogenesis in mature hybrids [55]. The meiosis of a cell containing two divergent genomes could undergo a number of deviations. Therefore, parthenogenesis might have evolved as a mechanism to prevent hybrid sterility. To assess genomic differences between the chromosomal structure of *D. mixta* and *D. valentini*, we referred to the obtained whole-genome alignment data (Supplementary Figure S4). The alignment plot demonstrated high structural resemblance between both genome assemblies. Synteny analysis also showed high collinearity between the corresponding chromosomes of the two *Darevskia* species (Figure 1). Our results are concordant with genomic analyses of *Aspidoscelis* reptiles that gave rise to the hybrid unisexual lineages [25]. In addition, whole-genome alignment data confirmed the similarity between the *Darevskia* assemblies and that of *L. agilis* (Supplementary Figures S2 and S4).

Despite structural conservation, all three genome assemblies bore a number of variations. Synteny analysis revealed multiple inversions and translocations between the homologous chromosomes. Genomic variations were identified in other parthenogenetic species [29,30,78,79], where hybridization between divergent but karyotypically similar species precedes the switch to unisexuality. Therefore, we assume that the observed divergent regions between the genome assemblies of *Darevskia* species might be associated with meiosis disruption in their hybrids. Comparison with a quality genome sequence of the unisexual progenitor may provide even more information on genomic regions possibly associated with the transition to parthenogenesis.

## 5. Conclusions

The transition to the parthenogenetic mode of reproduction is a rare phenomenon, yet its molecular genetic basis in *Darevskia* remains unknown. To identify the underlying molecular mechanisms of this switch, comprehensive and high-quality genomic resources should be created. In this study, we obtained reference-scaffolded chromosome-level genome assemblies of *D. mixta* and *D. valentini*—the parental species for parthenogenetic *D. armeniaca*. The assembled genomes are structurally similar, but include multiple inversions and translocations. The revealed architecture, repetitive landscape, and structural variation in these assemblies form a solid foundation for future research on parthenogenesis, sex chromosome evolution, and population genomics in the genus *Darevskia*. The created platform is useful in comparing the genomes of sexual and parthenogenetic *Darevskia* lizards, studying sex-determination loci and possible structural variants associated with reproductive mode, and analyzing gene expression and molecular pathways related to parthenogenesis.

## Figures and Tables

**Table 1:** QUAST and BUSCO statistics for *D. mixta* genome assemblies.

Tools for Assembly and Improvement	Total Length, Gb	Number of Contigs	Max Contig Length, Mb	N50, Mb	L50	BUSCO v5.8.3 (Sauropsida_odb10) *		
						C, %	D, %	M, %
Verkko	3.39	50,700	2.45	0.15	5575	96.8	73.5	2.3
HiFiasm	1.48	555	68.45	28.07	18	97.7	3.1	2.0
HiFiasm, Kraken	1.41	243	68.45	30.32	17	97.7	3.2	2.0
HiFiasm, Kraken, Pilon	1.41	243	68.33	30.28	17	97.8	3.2	1.9
HiFiasm, Kraken, Pilon, Seqkit	1.40	104	68.33	30.30	17	97.7	3.0	2.0

The lower the number of contigs and L50, and the higher the N50, the greater the assembly contiguity. * BUSCO—percentages of single-copy orthologues from the sauropsida_odb10 database found in the analyzed genome assemblies: C—complete; D—duplicated; M—missing.

**Table 2:** QUAST and BUSCO statistics for *D. valentini* genome assemblies.

Tools for Assembly and Improvement	Total Length, Gb	Number of Contigs	Max Contig Length, Mb	N50, Mb	L50	BUSCO v5.8.3 (Sauropsida_odb10) *		
						C, %	D, %	M, %
HiFiasm	1.47	630	43.42	10.85	41	97.6	4.1	2.1
HiFiasm, Seqkit	1.44	306	43.42	11.11	39	97.3	3.1	2.3

The lower the number of contigs and L50, and the higher the N50, the greater the assembly contiguity. * BUSCO—percentages of single-copy orthologues from the sauropsida_odb10 database found in the analyzed genome assemblies: C—complete; D—duplicated; M—missing.

## Data Availability

The original data presented in the study are openly available in the NCBI database under the BioProject accession number PRJNA1396639.

## Supplementary Materials

The following supporting information can be downloaded at https://www.mdpi.com/article/10.3390/ani16203311/s1. Table S1: *Darevskia mixta* contig positions in the reference assembly of *Lacerta agilis*; Table S2: *Darevskia valentini* contig positions in the reference assembly of *Lacerta agilis*; Figure S1: Whole-genome alignment of the obtained unscaffolded (a) *D. mixta* genome assembly (vertical axis) to the genome assembly of *L. agilis* (NCBI GCA_009819535.1) (horizontal axis) and (b) *D. valentini* genome assembly (vertical axis) to the genome assembly of *L. agilis* (NCBI GCA_009819535.1) (horizontal axis); Figure S2: Whole-genome alignment of the final (a) *D. mixta* genome assembly (vertical axis) to the genome assembly of *L. agilis* (NCBI GCA_009819535.1) (horizontal axis) and (b) *D. valentini* genome assembly (vertical axis) to the genome assembly of *L. agilis* (NCBI GCA_009819535.1) (horizontal axis); Figure S3: Assembly and analysis workflow for *Darevskia* genomes; Figure S4: Whole-genome alignment between the final assemblies of *D. mixta* (vertical axis) and *D. valentini* (horizontal axis).
